# Soybean Cultivar Breeding Has Increased Yields Through Extended Reproductive Growth Periods and Elevated Photosynthesis

**DOI:** 10.3390/plants14111675

**Published:** 2025-05-30

**Authors:** Hongbao Sun, Shuaijie Shen, Jingya Yang, Jun Zou, Matthew Tom Harrison, Zechen Wang, Jiaqi Hu, Haiyu Guo, Renan Caldas Umburanas, Yunlong Zhai, Xinya Wen, Fu Chen, Xiaogang Yin

**Affiliations:** 1 College of Agronomy and Biotechnology, China Agricultural University, Beijing 100193, China; sunhongbao6@163.com (H.S.); shuaijieshen@cau.edu.cn (S.S.); 18984562612@163.com (J.Y.); zoujun2018@163.com (J.Z.); 18801357909@163.com (Z.W.); 13354161038@163.com (J.H.); haiyughy@163.com (H.G.); wxya@cau.edu.cn (X.W.); chenfu@cau.edu.cn (F.C.); 2 Key Laboratory of Farming System, Ministry of Agriculture and Rural Affairs of China, Beijing 100193, China; 3 Tasmanian Institute of Agriculture, University of Tasmania, Newnham Drive, Launceston, TAS 7248, Australia; matthew.harrison@utas.edu.au; 4 Luiz de Queiroz College of Agriculture, University of São Paulo, Av. Pádua Dias, 11, Piracicaba 13418-900, SP, Brazil; rumbu@usp.br; 5 College of Agriculture, Tarim University, Alar 843300, China; zylzky@taru.edu.cn; 6 Key Laboratory of Genetic Improvement and Efficient Production for Specialty Crops in Arid Southern Xinjiang of Xinjiang Corps, Alar 843300, China

**Keywords:** soybean breeding, phenology, leaf area index, photosynthesis, yield components

## Abstract

Despite being one of China’s largest soybean (*Glycine max* L. Merr.) production areas, the Huanghuaihai Farming Region (HFR) has long been plagued by suboptimal yields. While cultivar development has contributed to yield gains in the past, whether such breeding will afford resilience under more adverse climatic conditions expected in future remains an open question. Here, we conducted two-year field experiments to contrast the growth and development of soybean cultivars released between 1960 and 2010 in the HFR. We found that cultivar breeding significantly influenced phenology, with contemporary cultivars having shorter and longer vegetative and reproductive growth phases, respectively. Grain filling duration of modern cultivars (LD11, HD14, JD21, and QH34) was 10 days longer than that of older cultivars (JX23 and WF7). Maturity height of modern cultivars decreased over time to a current value of ~80 cm, despite having higher leaf area index (LAI) and SPAD values compared with older cultivars during reproductive development. The quantum yield of electron transport in photosystem I, quantum yield of electron transport chain, photosynthetic performance index, stomatal conductance, net photosynthetic rate, and Rubisco activity of contemporary cultivars was stronger than those of older cultivars during grain filling. Prolonged grain filling duration, higher LAI, greater light interception, and stronger photosynthetic capacity evoked greater rates of grain filling, leading to higher grain weight, seed number, and yield. Genetic evolution of the cultivars over time, warmer conditions, and more precipitation together afforded longer reproductive stages. Our results indicate that yield gains have been realized primarily by cultivar breeding, and to a lesser extent, beneficial climate change. We highlight dynamic source/sink relationships underpinning the co-evolution of photosynthetic traits through soybean breeding, and provide practical advice to guide future breeding efforts.

## 1. Introduction

Soybean (*Glycine max* L. Merr.) is vital for contemporary food security, being widely grown across the world for oil and protein production [1]. As climate change has reduced the quantum and consistency of soybean production [2,3], significant efforts have been made to breed cultivars with high yield potential over the past six decades. For instance, average yields of soybean in the USA have increased by 70–90% compared with those from the 1960s [4], while yields in China increased by 15% since the year 2000 [5]. These improvements have been evoked through improved disease resistance, stress tolerance, and quality traits [6,7,8]. However, the physiological mechanisms underlying trait breeding during variety improvement remain uncertain due to the changing climate and development of new agronomic practices [9,10,11].

Crops with an optimal growing period tend to have higher resource-use efficiency (i.e., light, temperature, and water) [12,13]. Previous studies showed that global warming tends to increase crop development rates [14,15], causing shorter growth cycles [16,17]. For instance, the growing durations of soybean of China since 2000 have been truncated by 1–6 days, which significantly influences resource-use efficiency [18]. Plant phenotype also influences resource-use efficiency [19], with more recent cultivars displaying higher lodging resistance and shorter height [20,21]. Previous studies have shown that increased leaf photosynthesis has accompanied genetic yield improvement [22,23], underpinned by greater chlorophyll content and stronger sink capacity [24], although the extent to which these traits have changed over time is not well known.

The Huanghuaihai Farming Region (HFR), China’s second largest soybean production region [3], has long been characterized by low soybean yields (mean value of 1.7 Mg ha^−1^). Soybean germplasm has completed four large-scale cultivar replacements, contributing more than 200 varieties in the HFR over the last several decades [25]. Here, we selected seven widely cultivated soybean cultivars bred in the HFR from the 1960s to the 2010s. Using field experiments in the HFR during 2022 and 2023, our aim was to compare soybean cultivar responses to current climate conditions. The specific goals of this study were to: (1) contrast phenological durations of soybean cultivars from different decades, (2) evaluate the influence of cultivar breeding on leaf area index (LAI) and photosynthesis, and (3) dissect the influence of management, climate, and genotype on yield gains realized during the last 60 years.

## 2. Results

### 2.1. Changing Growing Periods over Several Decades

**Figure 1:**
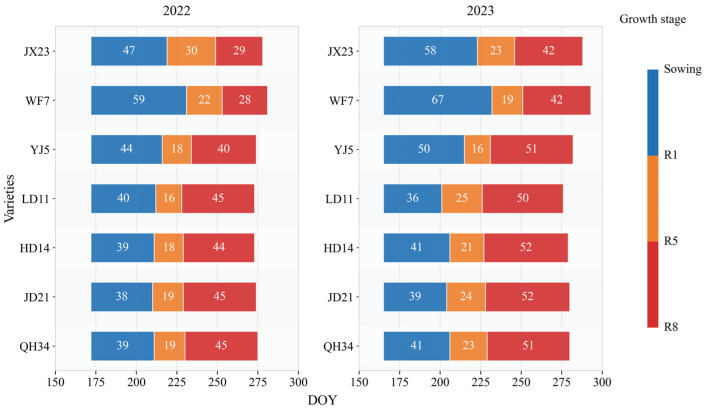
Change in length of growth period for soybean cultivars released during different decades measured in field experiments during 2022 and 2023. Blue, orange, and red bars represent length of the period of sowing–R1, R1–R5, and R5–R8, respectively.

We found that old cultivars had longer vegetative growth, with mean lengths of 53 and 63 days for JX23 and WF7, while the mean length for newer cultivars (LD11, HD14, JD21, and QH34) was less than 40 days (Figure 1). The reproductive period of older cultivars (i.e., JX23 and WF7) was shorter than that of modern cultivars (including LD11, HD14, JD21, and QH34), especially during grain filling (R5–R8; Figure 1). The mean filling duration (R5–R8) was 36 and 35 days for JX23 and WF7 (older cultivars), while for modern cultivars it was 48, 48, 49, and 48 days for LD11, HD14, JD21, and QH34. The year 2023 saw longer reproductive periods, likely due to greater rainfall at the same period in 2022 (Figure 1).

### 2.2. Influence of Cultivar Breeding on Plant Height and Leaf Area Index

**Figure 2:**
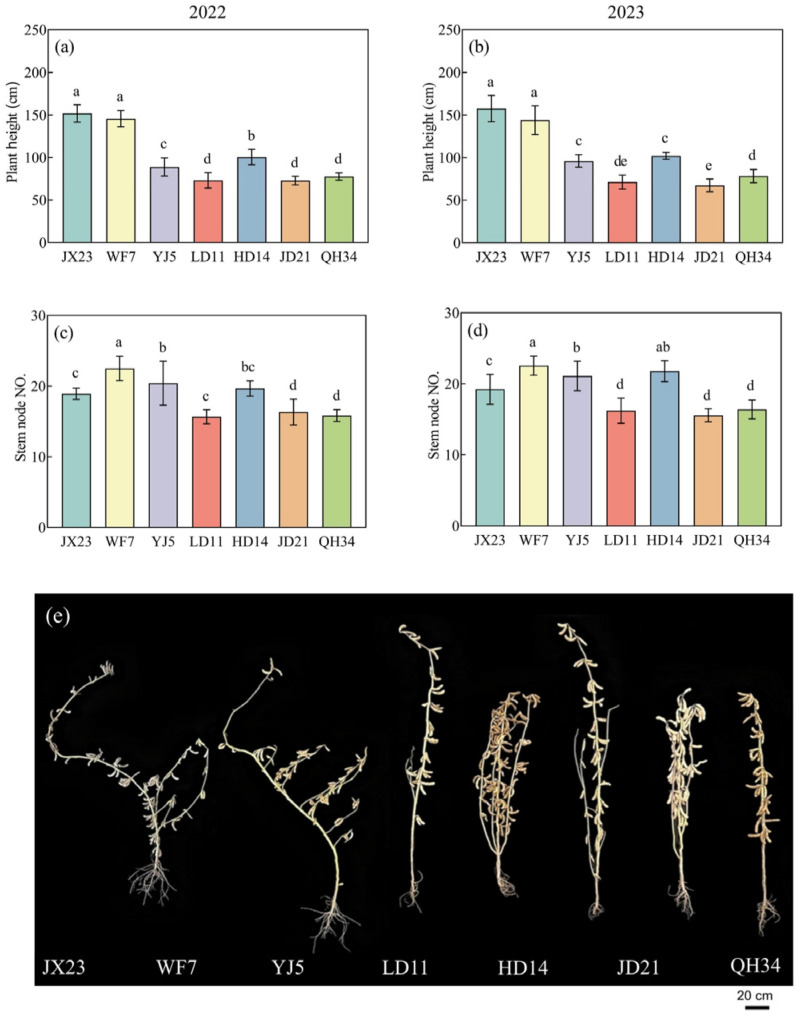
Plant height (**a**,**b**), and numbers of main stem nodes (**c**,**d**) of soybean cultivars grown in field experiments during 2022 and 2023, and (**e**) photo of plant phenotypes at the R8 stage in 2023. Error bars show standard error; different lowercase letters above bars indicate significant differences between cultivars (*p* < 0.05).

Plant height decreased with cultivar year of release. The height of older cultivars JX23 and WF7 was 155 and 145 cm, respectively (Figure 2), shorter than LD11, JD21, and QH34 (modern cultivars) with a mean value of 74 cm. Greater height led to more stem biomass, increasing the risk of lodging (Figure 2).

**Figure 3:**
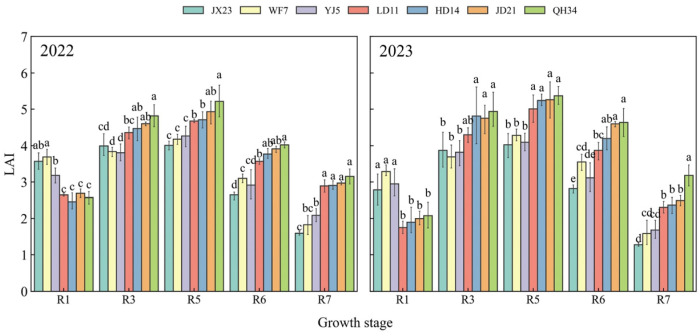
Leaf area index (LAI) of soybean cultivars released over several decades and grown in field experiments during 2022 and 2023. Different lowercase letters indicate significant differences between cultivars at the same growth stage (*p* < 0.05).

Maximum LAI values were observed at R5 for all cultivars (Figure 3). Older cultivars (JX23, WF7, and YJ5) had a higher LAI than modern cultivars (LD11, HD14, JD21, and QH34) at flowering (R1), and a lower LAI afterwards (Figure 3). The LAI of modern cultivars (LD11, HD14, JD21, and QH34) increased at a greater rate from R1 to R5 than that of older cultivars (JX23, WF7, and YJ5). Further, the LAI of modern cultivars (LD11, HD14, JD21, and QH34) during R5~R7 decreased less (40–53%) than that of older cultivars (JX23, WF7, and YJ5). Modern cultivars exhibited an ideal “early control, mid-term peak, late maintenance” pattern in LAI dynamics from R1 to R7, particularly maintaining a high LAI during the critical grain filling periods (R5–R7; Figure 3).

### 2.3. Impact of Plant Breeding on Relative Chlorophyll Content and Photosynthetic Traits

**Figure 4:**
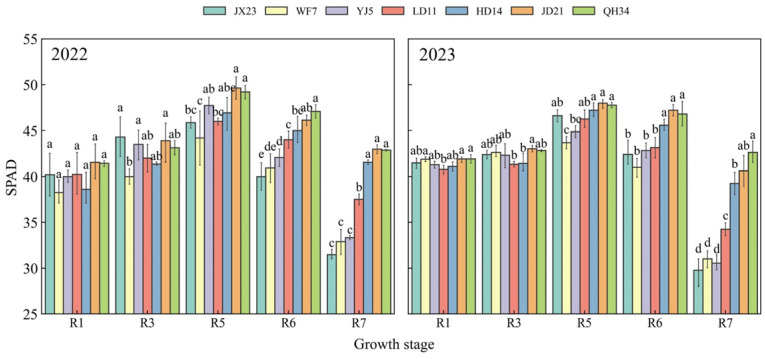
SPAD values (relative chlorophyll content) of soybean cultivars released during different decades and grown in field experiments during 2022 and 2023. Different lowercase letters indicate significant differences between cultivars at the same growth stage (*p* < 0.05).

Relative chlorophyll content (SPAD values) were similar at R1 (Figure 4). SPAD values of older cultivars (JX23, WF7, YJ5) peaked at R5 and decreased rapidly thereafter, with an average reduction of 32–36% from R5 to R7. Modern cultivars (LD11, HD14, JD21, and QH34) peaked at R5 and had a strong maintenance ability from R6 to R7. From R5 to R7, the SPAD of modern cultivars (LD11, HD14, JD21, and QH34) only decreased by 11–15%, while that of older cultivars (JX23, WF7, and YJ5) decreased at greater rates. Mean SPAD was 31, 32, and 32 for older cultivars (JX23, WF7, and YJ5) and 36, 40, 42, and 43 for modern cultivars (LD11, HD14, JD21, and QH34) at R7 (Figure 4). This high peak, slow decline characteristic in chlorophyll content of modern cultivars synergizes with their LAI dynamics, collectively realizing a longer, greener LAI, allowing longer seasonal light interception, photosynthesis, and growth.

**Figure 5:**
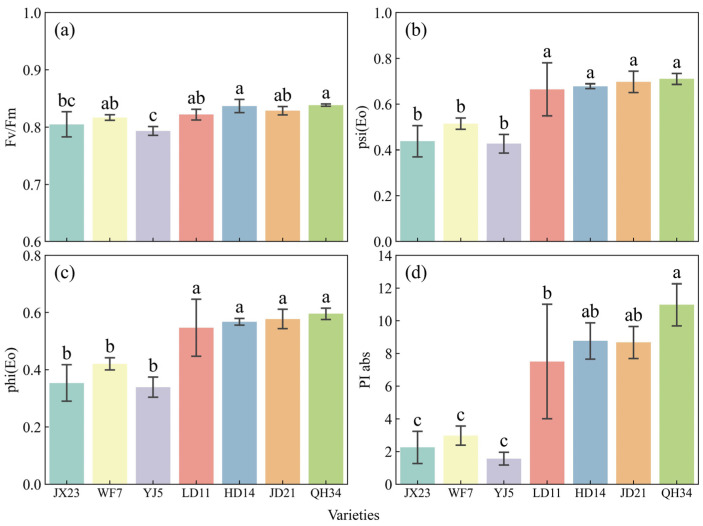
Photosystem electron transport efficiency measured at R5 in 2023 for soybean cultivars released across several decades. (**a**) Maximum quantum efficiency of photosystem II (Fv/Fm). (**b**) Quantum yield of electron transport in photosystem I [psi(Eo)]. (**c**) Quantum yield of electron transport chain [phi(Eo)]. (**d**) Photosynthetic performance index (PIabs). Error bars show standard error; different lowercase letters indicate significant differences between cultivars (*p* < 0.05).

Cultivar breeding increased photosynthetic electron transport efficiency (Figure 5). The maximum quantum efficiency of photosystem II (Fv/Fm) increased from older cultivars (JX23, WF7, YJ5) at 0.79–0.82 to modern cultivars (HD14, JD21, QH34) at 0.83–0.84. The quantum yield of photosystem I [psi(Eo)] in the electron transport chain showed a pronounced increase, rising from older cultivars (0.43–0.52) to modern cultivars (0.68–0.71). Similarly, quantum yield [phi(Eo)] also exhibited an upward trend, increasing from 0.34–0.42 in older cultivars to 0.57–0.60 in modern cultivars. Performance Index (PIabs) showed the most notable change, increasing from 2.25 in the 1960s variety JX23 to 10.98 in the 2010s variety QH34, a remarkable 387% gain (Figure 5).

**Figure 6:**
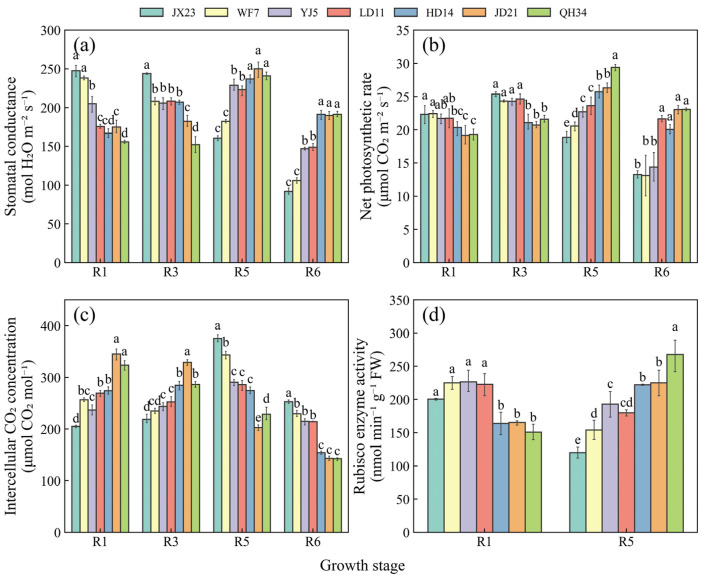
Dynamics of stomatal conductance (**a**), net photosynthetic rate (**b**), intercellular CO_2_ concentration (**c**), and Rubisco activity (**d**) at the reproductive stage in 2023 for soybean cultivars released during different decades. Data are presented as mean ± standard error; different letters indicate significant differences between cultivars at the same growth stage (*p* < 0.05).

Stomatal conductance and net photosynthetic rate showed similar trends during the reproductive period between different cultivars, both of which were higher for the older cultivars at R1 and R3, declining rapidly at R5 and R6 (Figure 6a,b). Conversely, stomatal conductance and net photosynthetic rate were much lower at R1 for the modern cultivars; the maximum values were observed at R5 and they were relatively high at R6 (Figure 6a,b). Intercellular CO_2_ concentration increased from R1 to R5, and declined at R6 for older cultivars (i.e., JX23 and WF7). In contrast, the same variable for modern cultivars decreased from R1 to R6 (i.e., JD21 and QH34) (Figure 6c). Rubisco activity was higher for older cultivars at R1 but declined rapidly at R5, while the reverse trend was true for modern cultivars (Figure 6d).

### 2.4. Variation in Grain Filling and Yield Component over Time

**Figure 7:**
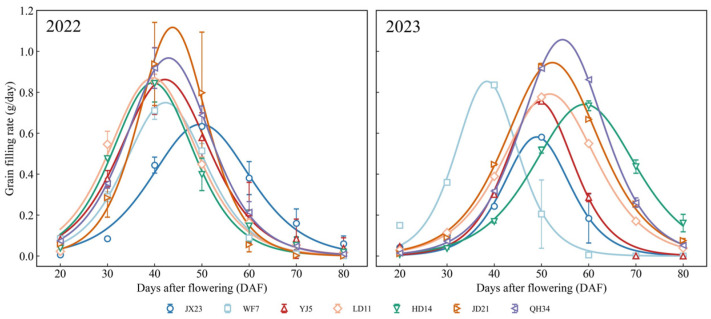
Dynamics of grain filling rate for soybean cultivars from different decades grown in field experiments during 2022 and 2023. Colored curves represent different cultivars. Error bars indicate standard error.

Grain filling characteristics changed with the release of new cultivars over time (Figure 7). Peak grain filling was attained 30–40 days after flowering for older cultivars (JX23 and WF7). The maximum filling rate of modern cultivars (2000–2010) was significantly higher than that of older cultivars; the 40-day filling rate of JD21 and QH34 was 109–111% higher than that of JX23 in 2022. Modern cultivars maintained higher grain filling rate with a longer period and a higher grain filling rate 50–60 days after flowering. These changes indicate that significant progress has been achieved in optimizing the growth period through breeding, especially by shortening the vegetative period and extending the grain filling period.

The mean yield was 1581 and 1791 kg ha^−1^ for older cultivars (JX23 and WF7), which was much lower than that of modern cultivars (JD21 and QH34) with mean values of 2862 and 3226 kg ha^−1^ (Table 1) during the experimental period. The low yield of older cultivars was driven by smaller 100-grain weight, pod number per plant, and seed number per plant (Table 1). Conversely, higher 100-grain weight, pod number per plant, and seed number per plant led to the high yield of modern cultivars (e.g., JD21 and QH34). The ANOVA indicated that variety had a significant effect on all traits. Year primarily affected 100-grain weight, pod number, and seed number (*p* < 0.01). Interaction between variety and year had a significant impact on yield and yield components (*p* < 0.05). These results suggest that modern breeding has not only enhanced yield potential but also improved varietal stability (Table 1).

### 2.5. Determinants of Soybean Yield Formation

**Figure 8:**
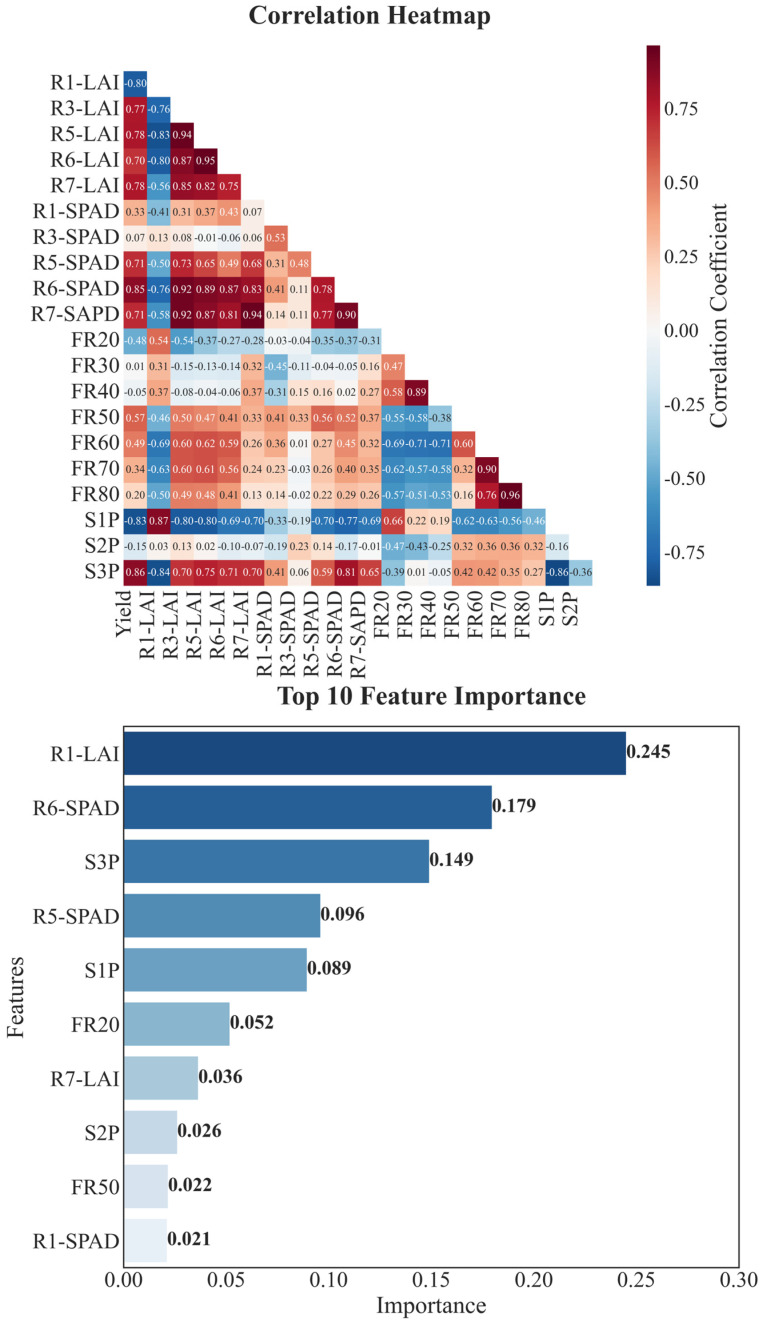
Correlation and importance analysis of soybean yield formation characteristics. Upper panel shows correlation heatmap between different characteristics, where darker colors indicate stronger correlations (red for positive, blue for negative). Lower panel shows feature importance ranking based on random forest algorithm. S1P, S2P, and S3P represent proportion of total growth period for Sowing–R1, R1–R5, and R5–R8 stages, respectively; R1-LAI, R3-LAI, R5-LAI, R6-LAI, and R7-LAI represent leaf area index across growth stages; R1-SPAD, R3-SPAD, R5-SPAD, R6-SPAD, and R7-SPAD represent relative chlorophyll content across growth stages.

Multidimensional trait analysis revealed that soybean yield formation was significantly influenced by growth period configuration, leaf functional traits, and grain filling processes. Correlation analysis showed that the proportion of the growth period during R5–R8 (S3P) had the strongest positive correlation with yield (r = 0.861), indicating that late growth development plays a crucial role in yield formation. In terms of leaf functional traits, SPAD values during R6 were significantly positively correlated with yield (r = 0.854). LAI during R5 and R7 and R3 also showed strong positive correlations with yield (correlation coefficients of 0.782, 0.779, and 0.776, respectively), suggesting that maintaining high photosynthetic performance and appropriate population structure is essential for yield formation (Figure 8).

Random forest feature importance analysis identified LAI during R1, SPAD values during R6, and S3P as the most important factors influencing yield, with their cumulative importance exceeding 50% (Figure 8). This indicates that high-yield soybean formation depends on an appropriate LAI during early growth (R1) to lay the foundation for later growth, maintaining a high LAI from R3 to R5 to ensure strong population photosynthetic capacity, high SPAD values during R6 to support grain filling, and a well-configured growth period during R5–R8 to ensure sufficient time for yield formation. Simultaneous optimization of these traits through modern breeding with stability across variable climatic conditions has effectively driven the significant yield gains measured here.

## 3. Discussion

### 3.1. Influence of Cultivar Breeding on Soybean Phenology

**Figure 9:**
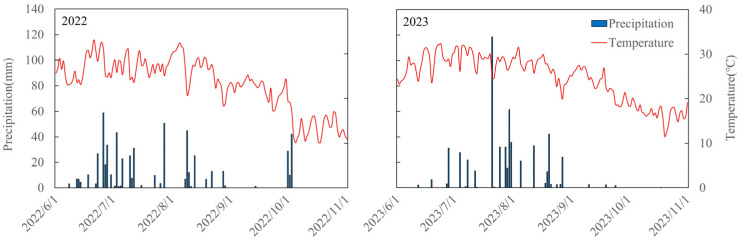
Daily mean temperature (red lines) and precipitation (blue bars) from 1 June to 31 October in 2022 and 2023.

**Figure A1:**
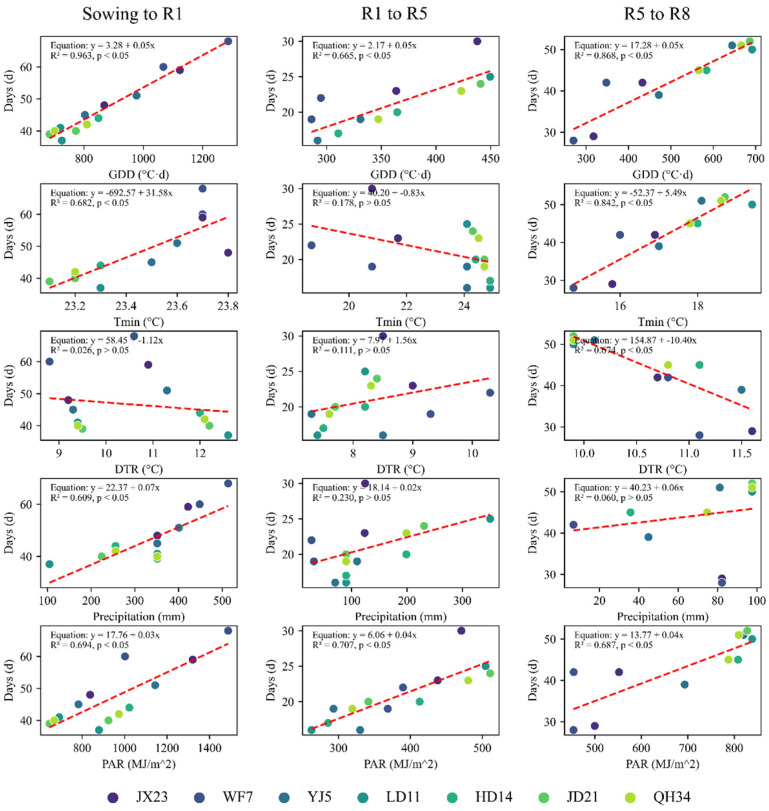
Correlation analysis between the duration of different growth periods and climatic factors (2022–2023). The *y*-axis “Days” represents the length of each growth period, including sowing to beginning bloom (Sowing–R1, days from sowing to 50% of plants flowering), beginning bloom to beginning seed (R1–R5, days from beginning bloom to 50% of plants beginning seed development), and beginning seed to full maturity (R5–R8, days from beginning seed development to 95% of plants reaching maturity). The *x*-axis shows climatic factors during each growth period, including growing degree days (GDDs, °C·d), minimum temperature (Tmin, °C), maximum temperature (Tmax, °C), diurnal temperature range (DTR, °C), precipitation (mm), and accumulated photosynthetically active radiation (Accumulated PAR, MJ/m^2^). Different colored dots represent different soybean cultivars (JX23, WF7, YJ5, LD11, HD14, JD21, and QH34), and red dashed lines indicate linear regression. The regression equation (y = ax + b), coefficient of determination (R^2^), and significance level (*p* < 0.05) are shown in the upper right corner of each subplot.

**Figure A2:**
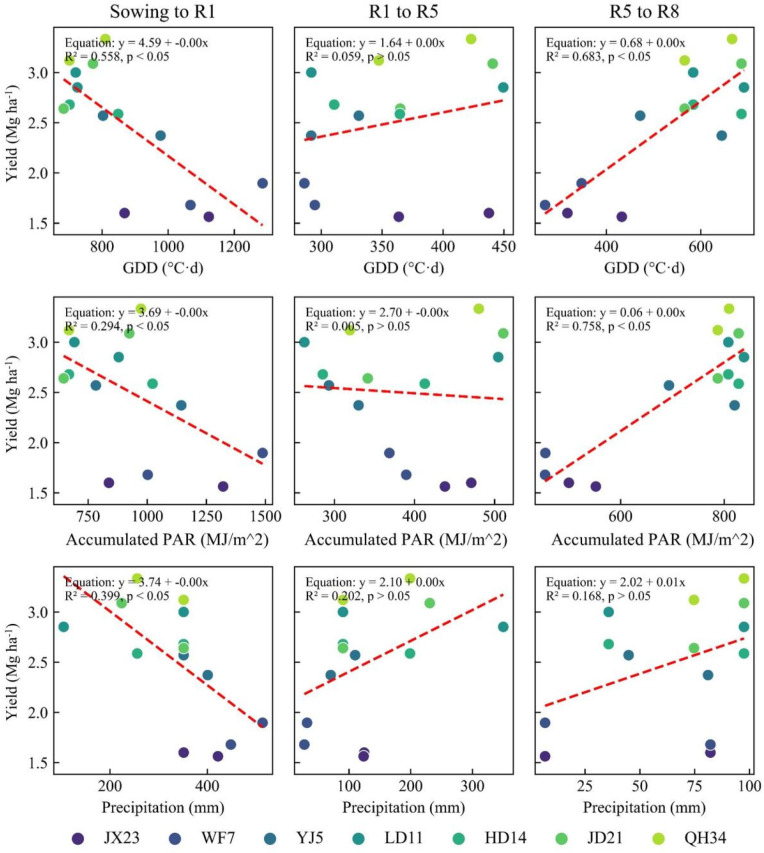
Correlations between GDDs, cumulative solar radiation, cumulative precipitation, and yield at each growth stage during the experimental period.

Soybean phenology has adapted to the changing climate and cultivar breeding in the HFR (Figure 1 and Figure 9), evidenced by shortened vegetative growth period and prolonged reproductive growth period. These changes were partly driven by increasing temperature and greater crop development that truncated vegetative growth durations [26,27]. Similar results were obtained in China’s national soybean cultivar during 2006 and 2020 [5]. While earlier flowering impairs yield potential if thermal time remains unchanged [10,28], reduced vegetative growth and longer grain filling durations associated with newer cultivars improved resource distribution, with more heat and solar radiation occurring during grain filling [29]. Modern cultivars were also better adapted to low-temperature exposure from R5 to R8, which was particularly beneficial for earlier and longer grain filling (Figure A1 and Figure A2). Previous studies have shown that sensitivity to temperature varies across developmental stages, influencing the formation of varietal adaptability [30,31]. More stable responses to warmer temperature observed in modern cultivars are likely the result of long-term selection targeting stage-specific responses during breeding. Long-term analysis via modelling would be an appropriate framework for extrapolating the field results we obtained here [32,33]; such an analysis would allow longitudinal assessment of climate relative to cultivar breeding, including effects of extreme weather events, such as extreme cold, drought, and/or heat waves [34].

### 3.2. Effects of Cultivar Breeding on Phenotype and Photosynthesis

Plant height decreased along with soybean cultivar breeding in the HFR during the past 60 years. In general, higher soybean yield is related to planting density, while higher plant density can cause lodging and yield loss [35]. Moreover, warmer conditions and high precipitation in summer are conducive to prolific soybean growth, which also promotes lodging risk [25]. Our results suggest that modern cultivars are better adapted to local weather conditions and planting patterns compared to older cultivars (Figure 2). While dwarfing is conducive to higher planting density [7], benefitting yield [36,37], increased planting density may reduce the availability of heat and radiation per plant [38]. A longer grain filling period and the stability of LAI and SPAD in the later growth stage of modern cultivars can delay leaf senescence and maintain leaf function [39], improving the productivity of individual plants and crop yield.

Cultivar breeding increased photosynthetic capability (Figure 5 and Figure 6), similar to previous research in Northeast China [22]. Higher photosynthetic rates of modern cultivars at R5 may be associated with higher Rubisco activity [40], which in turn requires more nitrogen [41,42]. Generally, modern cultivars were mostly bred under high nitrogen fertilizer conditions, while older cultivars were bred under low nitrogen fertilizer conditions. Higher leaf nitrogen of modern cultivars compared with early cultivars [43] was conducive to higher Rubisco activity [44]. High LAI and SPAD can ensure higher light energy conversion efficiency [24,45]. These factors together contributed to the superior performance of modern variety sources, ensuring uniform distribution of leaf nitrogen and sufficient accumulation of dry matter. In addition, the sink capacity of modern soybean cultivars has been significantly enhanced along with breeding development through grain weight [46,47]. The combined availability of sufficient sources and sinks of modern cultivars were more conducive to higher yields than older cultivars.

### 3.3. Implications and Perspectives

Cultivar breeding contributed to high soybean yield through reduced vegetative growth stage and extended grain filling, with more heat and radiation resources for better yield formation. Greater photosynthetic ability and longer photosynthetic time both promote grain filling, improving potential yield. Our results suggest that older soybean cultivars are not well adapted to current climate conditions, with newer cultivars having shorter height, less biomass in stems, stronger photosynthesis capability, and longer grain filling. These results are comparable to those in dual-purpose crops, where livestock defoliation of biomass during vegetative phases is conductive to a longer reproductive period, greater green area duration, greater photosynthesis post anthesis, and soil water conservation [48,49,50].

As we primarily focused on agronomic parameters, further measurements are necessary to fully dissect the effects of cultivar breeding on yield improvement under changing climate conditions. As well, using only two years of field experiments may limit the insights to bespoke weather conditions. Future studies could grow these cultivars over longer periods to ascertain the mechanism by which variety renewal drives yield improvement. These limitations could be overcome using modelling to dissect the influence of management from genotype and environment [11,34]. This modelling could also examine landscape-level influences, such as resource and infrastructure availability, as well as peer-to-peer learnings [28,51].

## 4. Materials and Methods

### 4.1. Experimental Site

Field experiments were conducted at the Wuqiao Experimental Station (37°41′ N, 116°37′ E) of China Agricultural University in 2022 and 2023. The site is a temperate monsoon climate zone, with an average annual precipitation of 400–700 mm, an average annual temperature of 12–14 °C, and a frost-free period of about 200 days [52]. Daily weather data, including maximum temperature, minimum temperature, mean temperature, rainfall, sunshine hours, and solar radiation, were obtained from the automatic weather station (ARN-X automatic weather monitoring station) at the experimental site during the experimental period. Higher precipitation with warm conditions was observed in 2023 (Figure 9). The soil physical and chemical properties of the 0–40 cm soil layer in the test site were classified as a sandy loam according to the classification standard of soil texture of the United States Department of Agriculture, with relatively consistent fertility across the site [53].

### 4.2. Experimental Design

Seven representative soybean cultivars that were widely planted in the HFR since the 1960s were adopted here [1], including Juxuan 23 (JX23) from the 1960s, Wenfeng 7 (WF7) from the 1970s, Yuejin 5 (YJ5) from the 1980s, Ludou 11 (LD11) from the 1990s, Hedou 14 (HD14) from the 2000s, Jidou 21 (JD21) from the 2010s, and Qihuang 34 (QH34) from the 2010s. Each treatment was repeated 4 times with a plot size of 21 m^2^; sowing density was 240,000 plants per hectare with row spacing of 40 cm and plant spacing of 15 cm. During the experimental period, two seeds were sown in one hole; plants were thinned at seedling stage according to target plant population. In 2022, all cultivars were sown on 21st June and harvested on 15 October; in 2023, all cultivars were sown on 14 June and harvested 20 October, respectively. Fertilizers were applied before sowing in both years at 20 kg ha^−1^ N, 60 kg ha^−1^ P_2_O_5_, and 20 kg ha^−1^ K_2_O, respectively. Timely weed and pest control was conducted throughout the growth period.

### 4.3. Measurements

#### 4.3.1. Phenology

Phenology was monitored according to developmental criteria, including the seedling stage (VE), beginning of flowering (R1), full flowering (R2), beginning pod filling (R3), beginning grain filling (R5), drumming (R6), early maturation (R7), and maturity (R8) [27].

#### 4.3.2. Leaf Area Index, Relative Chlorophyll Content (SPAD), and Photosynthesis

Leaf area was measured using a canopy analyzer (LI-3100C leaf area analyzer, LI-COR Environmental, Lincoln, NE, USA). SPAD values of three inverted leaves on the main stem were measured using a portable chlorophyll analyzer (SPAD502, Konica Minolta, Tokyo, Japan) at R1, R3, R5, R6, and R7 in 2022 and 2023, respectively. Each treatment was measured 10 times. A Li-6400 portable photosynthesis system (LI-COR Inc., Lincoln, NE, USA) was used to measure photosynthetic parameters of soybean leaves at the R1, R3, R5, and R6 stage of each soybean cultivar. Net photosynthetic rate (Pn), stomatal conductance (Gs), and intercellular CO_2_ concentration (Ci) were measured under a light intensity of 600 μmol m^−2^ s^−1^, temperature of 25 °C, and a CO_2_ concentration of 460 (μmol mol^−1^) from 9:00 to 11:00. In the R5 stage of each cultivar, chlorophyll fluorescence parameters of leaves were determined using a Handy PEA plant efficiency analyzer (Hansatech Company products, Hansatech Instruments Ltd., King’s Lynn, Norfolk, UK), which included determination of PSII photochemical efficiency and Fv/Fm value [54,55,56].

At R1 and R5 in 2023, the penultimate fully unfolded leaf of the main stem was taken and frozen in liquid nitrogen, then stored in an ultra-low-temperature refrigerator at −80 °C for Rubisco enzyme activity determination following the plant ribulose-1,5-bisphosphate carboxylase (Rubisco) kit produced by Suzhou Grease Biotechnology Co., Ltd., Suzhou, China.

#### 4.3.3. Grain Filling Rate

Grain filling rate was measured in 2022 and 2023 for each cultivar. Plants were tagged at R1 and sampled at R6. Sampling strips of each community were symmetrically sampled according to the edge row and the middle row. Every 7 days, 6 plants were selected, seeds were peeled off and mixed, and 100 grains were oven dried. Taking the number of days after flowering (*t*) as the independent variable, the 100-grain weight was measured each time as the dependent variable (*W*), with grain growth fitted with the logistic equation: *W* = *A*/(1 + *B* · e^−*C*·*t*^), where in the formula: *t* is the number of days after flowering; *W* is the 100-grain weight; *A* is a theoretical maximum 100-grain weight; *B* and *C* are parameters determined by curvature.

#### 4.3.4. Yield and Yield Components

Four rows in the center of each plot with an area of 12 square meters were harvested to measure soybean yield. At maturity (R8), ten plants were randomly chosen from each plot. Plant height, stem diameter, number of branches, number of nodes, number of pods per plant, number of grains per plant, number of grains per pod, and 100-grain weight were measured.

#### 4.3.5. Data Analysis

ANOVA was conducted after confirming that normality and heterogeneity assumptions were satisfied. ANOVA and the least significant difference (LSD) test were conducted using Python (3.8, Python Software Foundation). Random forest was used to evaluate the importance of factors including growth period, grain filling rate, and physiological indices affecting soybean yield [57].

## 5. Conclusions

We found that soybean cultivar breeding contributed significantly to yield gains since 1960 in the HFR. This improvement is likely underpinned by extended grain filling and reduced vegetative growth stage of modern cultivars (LD11, HD14, JD21, and QH34) compared with older cultivars (JX23 and WF7). Modern cultivars (LD11, HD14, JD21, and QH34) also had larger LAI and higher SPAD than that of older cultivars (JX23 and WF7), improving quantum yield of electron transport in photosystem I, quantum yield of electron transport chain, photosynthetic performance index, stomatal conductance, net photosynthetic rate, and Rubisco activity of modern cultivars. Extended grain filling duration, higher LAI, and stronger photosynthetic capacity evoked greater grain filling, leading to higher grain weight and seed number, and yield. At the same time, propitious changes in climate (warmer temperatures and improved rainfall during grain filling) were conducive to longer reproductive duration and yield. Our results indicate that yield gains have been afforded primarily by cultivar breeding selection, but also beneficial climate change.

## Figures and Tables

**Table 1:** Yield and yield components for soybean cultivars developed over six decades grown in field experiments during 2022 and 2023.

Year	Cultivar	Grain Yield (kg ha^−1^)	100-Grain Weight (g)	Pod Number (plant^−1^)	Seed Number (plant^−1^)	Seeds per Pod
2022	JX23	1580 ± 64 b	13.3 ± 0.0 f	50.4 ± 1.7 b	89.8 ± 5.8 b	1.8 ± 0.1 c
	WF7	1683 ± 326 b	15.6 ± 0.1 e	43.9 ± 0.7 c	89.3 ± 4.2 b	2.0 ± 0.1 bc
	YJ5	2570 ± 392 a	19.7 ± 0.3 d	46.5 ± 3.4 bc	95.9 ± 4.5 ab	2.1 ± 0.2 bc
	LD11	3004 ± 127 a	20.1 ± 0.1 c	54.7 ± 3.2 a	111.3 ± 14.7 a	2.0 ± 0.2 bc
	HD14	2678 ± 364 a	20.2 ± 0.2 c	45.3 ± 0.3 bc	101.4 ± 3.8 ab	2.2 ± 0.1 ab
	JD21	2636 ± 498 a	21.1 ± 0.1 b	47.3 ± 1.8 bc	100.2 ± 6.5 ab	2.1 ± 0.1 ab
	QH34	3119 ± 553 a	22.9 ± 0.2 a	47.2 ± 0.8 bc	112.8 ± 1.7 a	2.4 ± 0.0 a
2023	JX23	1563 ± 379 d	14.4 ± 0.1 e	52.3 ± 9.1 b	70.7 ± 5.8 bc	1.7 ± 0.1 c
	WF7	1899 ± 46 d	17.7 ± 0.1 d	44.0 ± 1.0 c	77.0 ± 7.5 c	2.0 ± 0.1 bc
	YJ5	2372 ± 198 c	19.9 ± 0.2 c	52.7 ± 4.0 b	82.0 ± 10.0 ab	2.2 ± 0.1 ab
	LD11	2850 ± 415 abc	20.1 ± 0.2 c	65.3 ± 4.9 a	103.3 ± 12.1 a	1.9 ± 0.1 bc
	HD14	2587 ± 609 bc	20.1 ± 0.1 c	55.0 ± 2.0 b	104.3 ± 9.0 a	2.3 ± 0.1 a
	JD21	3089 ± 435 ab	23.8 ± 0.4 b	49.3 ± 7.5 bc	97.0 ± 14.5 a	2.3 ± 0.2 a
	QH34	3333 ± 299 a	24.1 ± 0.1 a	46.3 ± 2.1 bc	96.3 ± 6.7 a	2.5 ± 0.1 a
ANOVA	Year (Y)	NS	***	**	***	NS
	Cultivars (V)	***	***	***	***	***
	Y * V	*	***	*	*	NS

Different letters indicate statistically significant difference between cultivars (*p* < 0.05), NS = No Significant; * *p* < 0.05; ** *p* < 0.01; *** *p* < 0.001.

## Data Availability

Data will be made available upon request.

## References

[1] Nie J.W., Zhou J., Zhao J., Wang X.Q., Liu K., Wang P.X., Wang S., Yang L., Zang H.D., Harrison M.T. (2022). Soybean Crops Penalize Subsequent Wheat Yield During Drought in the North China Plain. Front. Plant Sci..

[2] Zhao C., Liu B., Piao S.L., Wang X.H., Lobell D.B., Huang Y., Huang M.T., Yao Y.T., Bassu S., Ciais P. (2017). Temperature increase reduces global yields of major crops in four independent estimates. Proc. Natl. Acad. Sci. USA.

[3] Yang Y., Zou J., Huang W.H., Olesen J.E., Li W.J., Rees R.M., Harrison M.T., Feng B., Feng Y.P., Chen F. (2024). Drivers of soybean-based rotations synergistically increase crop productivity and reduce GHG emissions. Agric. Ecosyst. Environ..

[4] Westcott P., Hansen J. (2016). USDA Agricultural Projections to 2025.

[5] Zhang L., Zheng H.Y., Li W.J., Olesen J.E., Harrison M.T., Bai Z.Y., Zou J., Zheng A.X., Bernacchi C., Xu X.Y. (2023). Genetic progress battles climate variability: Drivers of soybean yield gains in China from 2006 to 2020. Agron. Sustain. Dev..

[6] Patil G., Mian R., Vuong T., Pantalone V., Song Q.J., Chen P.Y., Shannon G.J., Carter T.C., Nguyen H.T. (2017). Molecular mapping and genomics of soybean seed protein: A review and perspective for the future. Theor. Appl. Genet..

[7] Liu S.L., Zhang M., Feng F., Tian Z.X. (2020). Toward a “Green Revolution” for Soybean. Mol. Plant.

[8] Li M.W., Jiang B.J., Han T.F., Zhang G.H., Lam H.M. (2022). Genomic research on soybean and its impact on molecular breeding. Adv. Bot. Res..

[9] Wei W., Li Q.-T., Chu Y.-N., Reiter R.J., Yu X.-M., Zhu D.-H., Zhang W.-K., Ma B., Lin Q., Zhang J.-S. (2015). Melatonin enhances plant growth and abiotic stress tolerance in soybean plants. J. Exp. Bot..

[10] Muleke A., Harrison M.T., de Voil P., Hunt I., Liu K., Yanotti M., Eisner R. (2022). Earlier crop flowering caused by global warming alleviated by irrigation. Environ. Res. Lett..

[11] Phelan D.C., Harrison M.T., McLean G., Cox H., Pembletond K.G., Dean G.J., Parsons D., Richter M.E.D., Pengilley G., Hinton S.J. (2018). Advancing a farmer decision support tool for agronomic decisions on rainfed and irrigated wheat cropping in Tasmania. Agric. Syst..

[12] Beharav A., Hellier B. (2020). Bolting and flowering response ofLactuca georgica, a wild lettuce relative, to low temperatures. Am. J. Plant Sci..

[13] Egli D.B., Bruening W.P. (2001). Source-sink relationships, seed sucrose levels and seed growth rates in soybean. Ann. Bot-Lond..

[14] Sabbahi R. (2022). Effects of Climate Change on Insect Pollinators and Implications for Food Security—Evidence and Recommended Actions.

[15] Kistner E.J. (2017). Climate Change Impacts on the Potential Distribution and Abundance of the Brown Marmorated Stink Bug (Hemiptera: Pentatomidae) With Special Reference to North America and Europe. Environ. Entomol..

[16] Aggarwal P., Vyas S., Thornton P., Campbell B.M., Kropff M. (2019). Importance of considering technology growth in impact assessments of climate change on agriculture. Glob. Food Secur. Agric. Policy Econ. Environ..

[17] Muleke A., Harrison M.T., Eisner R., de Voil P., Yanotti M., Liu K., Yin X.G., Wang W.L., Monjardino M., Zhao J. (2022). Whole farm planning raises profit despite burgeoning climate crisis. Sci. Rep..

[18] Liu Y.J., Dai L. (2020). Modelling the impacts of climate change and crop management measures on soybean phenology in China. J. Clean. Prod..

[19] Liu K., Harrison M.T., Wang B., Yang R., Yan H.L., Zou J., Liu D.L., Meinke H., Tian X.H., Ma S.Y. (2022). Designing high-yielding wheat crops under late sowing: A case study in southern China. Agron. Sustain. Dev..

[20] Ustun A., Allen F.L., English B.C. (2001). Genetic progress in soybean of the US midsouth. Crop Sci..

[21] Liu G., Yang C., Xu K., Zhang Z., Li D., Wu Z., Chen Z. (2012). Development of yield and some photosynthetic characteristics during 82 years of genetic improvement of soybean genotypes in northeast China. Aust. J. Crop Sci..

[22] Jin J., Liu X.B., Wang G.H., Mi L., Shen Z.B., Chen X.L., Herbert S.J. (2010). Agronomic and physiological contributions to the yield improvement of soybean cultivars released from 1950 to 2006 in Northeast China. Field Crops Res..

[23] Morrison M.J., Voldeng H.D., Cober E.R. (1999). Physiological changes from 58 years of genetic improvement of short-season soybean cultivars in Canada. Agron. J..

[24] Koester R.P., Nohl B.M., Diers B.W., Ainsworth E.A. (2016). Has photosynthetic capacity increased with 80years of soybean breeding? An examination of historical soybean cultivars. Plant Cell Environ..

[25] Wang C.J., Wu T.T., Sun S., Xu R., Ren J.J., Wu C.X., Jiang B.J., Hou W.S., Han T.F. (2016). Seventy-five Years of Improvement of Yield and Agronomic Traits of Soybean Cultivars Released in the Yellow-Huai-Hai River Valley. Crop Sci..

[26] He L., Jin N., Yu Q. (2020). Impacts of climate change and crop management practices on soybean phenology changes in China. Sci. Total Environ..

[27] Zheng H.Y., Zhang L., Sun H.B., Zheng A.X., Harrison M.T., Li W.J., Zou J., Zhang D.T., Chen F., Yin X.G. (2024). Optimal sowing time to adapt soybean production to global warming with different cultivars in the Huanghuaihai Farming Region of China. Field Crops Res..

[28] Meier E.A., Thorburn P.J., Bell L.W., Harrison M.T., Biggs J.S. (2020). Greenhouse Gas Emissions from Cropping and Grazed Pastures Are Similar: A Simulation Analysis in Australia. Front. Sustain. Food Syst..

[29] Wang S., Liu S., Wang J., Yokosho K., Zhou B., Yu Y.-C., Liu Z., Frommer W.B., Ma J.F., Chen L.-Q. (2020). Simultaneous changes in seed size, oil content and protein content driven by selection of SWEET homologues during soybean domestication. Natl. Sci. Rev..

[30] Setiyono T.D., Weiss A., Specht J., Bastidas A.M., Cassman K.G., Dobermann A. (2007). Understanding and modeling the effect of temperature and daylength on soybean phenology under high-yield conditions. Field Crops Res..

[31] Kumagai E., Sameshima R. (2014). Genotypic differences in soybean yield responses to increasing temperature in a cool climate are related to maturity group. Agric. For. Meteorol..

[32] Tao R., Zhao P., Wu J., Martin N., Harrison M.T., Ferreira C., Kalantari Z., Hovakimyan N. Optimizing Crop Management with Reinforcement Learning and Imitation Learning. Proceedings of the Thirty-Second International Joint Conference on Artificial Intelligence, IJCAI 2023.

[33] Taylor C.A., Harrison M.T., Telfer M., Eckard R. (2016). Modelled greenhouse gas emissions from beef cattle grazing irrigated leucaena in northern Australia. Anim. Prod. Sci..

[34] Harrison M.T. (2021). Climate change benefits negated by extreme heat. Nat. Food.

[35] Li S.C., Sun Z.H., Sang Q., Qin C., Kong L.P., Huang X., Liu H., Su T., Li H.Y., He M.L. (2023). Soybean reduced internode 1 determines internode length and improves grain yield at dense planting. Nat. Commun..

[36] Morrison M.J., Voldeng H.D., Cober E.R. (2000). Agronomic changes from 58 years of genetic improvement of short-season soybean cultivars in Canada. Agron. J..

[37] Wilcox J.R. (2001). Sixty years of improvement in publicly developed elite soybean lines. Crop Sci..

[38] Zhang D.S., Sun Z.X., Feng L.S., Bai W., Yang N., Zhang Z., Du G.J., Feng C., Cai Q., Wang Q. (2020). Maize plant density affects yield, growth and source-sink relationship of crops in maize/peanut intercropping. Field Crops Res..

[39] Tang Y., Lu S.J., Fang C., Liu H., Dong L.D., Li H.Y., Su T., Li S.C., Wang L.S., Cheng Q. (2023). Diverse flowering responses subjecting to ambient high temperature in soybean under short-day conditions. Plant Biotechnol. J..

[40] Gago J., Daloso D.M., Carriquí M., Nadal M., Morales M., Araújo W.L., Nunes-Nesi A., Flexas J. (2020). Mesophyll conductance: The leaf corridors for photosynthesis. Biochem. Soc. Trans..

[41] Raven J.A. (2013). Rubisco: Still the most abundant protein of Earth?. New Phytol..

[42] Das A., Rushton P.J., Rohila J.S. (2017). Metabolomic Profiling of Soybeans (*Glycine max* L. Merr.) Reveals the Importance of Sugar and Nitrogen Metabolism under Drought and Heat Stress. Plants.

[43] Li D., Chen Z., Xu K., Zhang Z., Wu Z., Ji P., Zhang P. (2013). Changes of nitrogen content in leaf and its correlations with net photosynthetic rate of soybean cultivars released in different years. Chin. J. Oil Crop Sci..

[44] Li B., Gao K., Ren H., Tang W. (2018). Molecular mechanisms governing plant responses to high temperatures. J. Integr. Plant Biol..

[45] Hammad H.M., Chawla M.S., Jawad R., Alhuqail A., Bakhat H.F., Farhad W., Khan F., Mubeen M., Shah A.N., Liu K. (2022). Evaluating the Impact of Nitrogen Application on Growth and Productivity of Maize Under Control Conditions. Front. Plant Sci..

[46] Paul M.J., Watson A., Griffiths C.A. (2020). Linking fundamental science to crop improvement through understanding source and sink traits and their integration for yield enhancement. J. Exp. Bot..

[47] Slafer G.A., Foulkes M.J., Reynolds M.P., Murchie E.H., Carmo-Silva E., Flavell R., Gwyn J., Sawkins M., Griffiths S. (2023). A ‘wiring diagram’ for sink strength traits impacting wheat yield potential. J. Exp. Bot..

[48] Harrison M.T., Evans J.R., Dove H., Moore A.D. (2011). Recovery dynamics of rainfed winter wheat after livestock grazing 1. Growth rates, grain yields, soil water use and water-use efficiency. Crop Pasture Sci..

[49] Harrison M.T., Evans J.R., Dove H., Moore A.D. (2011). Recovery dynamics of rainfed winter wheat after livestock grazing 2. Light interception, radiation-use efficiency and dry-matter partitioning. Crop Pasture Sci..

[50] Harrison M.T., Evans J.R., Moore A.D. (2012). Using a mathematical framework to examine physiological changes in winter wheat after livestock grazing 2. Model validation and effects of grazing management. Field Crops Res..

[51] Shahpari S., Allison J., Harrison M.T., Stanley R. (2021). An Integrated Economic, Environmental and Social Approach to Agricultural Land-Use Planning. Land.

[52] Zhang D.T., Shen S.J., Bai Z.Y., Harrison M.T., Liu K., Rees R.M., Topp C.F.E., Zou J., Yang Y.H., Song Z.W. (2025). Optimizing N rate in wheat-maize rotation to match long-term and inter-seasonal N turnover for high yield and sustainability using STICS. Field Crops Res..

[53] Shen S.J., Feng B., Zhang D.T., Zou J., Yang Y.H., Rees R.M., Topp C.F.E., Hu S.Y., Qiao B.W., Huang W.H. (2025). Optimizing N applications increases maize yield and reduces environmental costs in a 12-year wheat-maize system. Field Crops Res..

[54] Tacarindua C.R.P., Shiraiwa T., Homma K., Kumagai E., Sameshima R. (2013). The effects of increased temperature on crop growth and yield of soybean grown in a temperature gradient chamber. Field Crops Res..

[55] Hu Y., Zhu Y., Yang Y., Zou J., Chen F., Yin X. (2019). Changes of the planting structure of major food and oil crops in China from 1951 to 2015. J. China Agric. Univ..

[56] Ergo V.V., Veas R.E., Vega C.R.C., Lascano R., Carrera C.S. (2021). Leaf photosynthesis and senescence in heated and droughted field-grown soybean with contrasting seed protein concentration. Plant Physiol. Biochem..

[57] Yang L., Song W.W., Xu C.L., Sapey E., Jiang D., Wu C.X. (2023). Effects of high night temperature on soybean yield and compositions. Front. Plant Sci..

